# Screening of the Pathogen Box reveals new starting points for anti-trypanosomal drug discovery†
†Electronic supplementary information (ESI) available. See DOI: 10.1039/c8md00319j


**DOI:** 10.1039/c8md00319j

**Published:** 2018-10-26

**Authors:** Clinton G. L. Veale, Heinrich C. Hoppe

**Affiliations:** a School of Chemistry and Physics , Pietermaritzburg Campus , University of KwaZulu-Natal , Private Bag X01 , Scottsville , 3209 , South Africa . Email: VealeC@ukzn.ac.za ; Tel: +27 33 260 6365; b Department of Biochemistry and Microbiology , Rhodes University , Grahamstown , 6140 , South Africa . Email: H.Hoppe@ru.ac.za ; Tel: +27 46 603 8262

## Abstract

This study aimed to uncover new starting points for anti-trypansomal drug discovery through the screening of the Pathogen Box against *Trypanosoma brucei brucei*. Our study identified compounds **35**, **39**, **46**, **53** and **56** whose activity and selectivity highlighted them as promising candidates with potential for further study and optimisation.

## Abstract 2

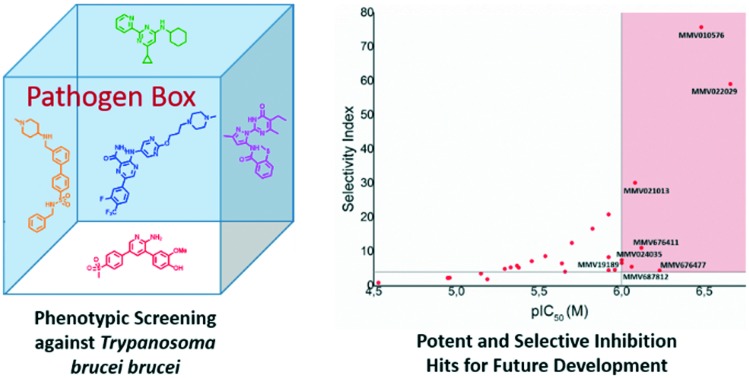
This study has identified several compounds with potential for repurposing against *Trypanosoma brucei*.

## Introduction

Human African trypanosomiasis (HAT), commonly referred to as sleeping sickness whose causative agent is *Trypanosoma brucei* (*T.b.*), impacts roughly 70 million people living in sub-Saharan Africa, affecting these populations from both a health and economic perspective.^1^,^2^ With no current vaccine and limited access to appropriate facilities for early diagnosis, coupled to treatment regimens which are limited in their scope and efficacy,^3^,^4^ the importance of stimulating new drug discovery programs through cost effective means is of grave importance.^5^ HAT is caused by the *T.b.* sub-species *T.b. rhodesiense* and *T.b. gambiense* respectively, while African animal trypanosomiasis (AAT) is typically caused by the *T.b. brucei* subspecies.^6^,^7^ While *T.b.* sub-species infections are clinically distinct, they share large similarities in both their molecular and cellular biology,^8^ which has allowed for *T.b. brucei* to be used as a competent proxy for the identification of hits and leads for HAT.^9^–^12^ The Pathogen Box like the Malaria Box represents a 400 strong library of compounds with demonstrated biological activity against a range of neglected tropical diseases,^13^ which has formed the basis of numerous screening campaigns to identify chemical starting points for hit and lead optimisation campaigns.^14^–^16^ Van Voorhis *et al.*^13^ reported a meta-analysis of multiple screens of the Malaria Box, which included anti-trypanosomal hit compounds. Similarly, in our efforts to identify new starting points for anti-trypanosomal drug discovery, we screened the Pathogen Box against *T.b. brucei* and identified a handful of compounds with encouraging and selective *in vitro* activity. During the process of our screening campaign, we were alerted to a study by Duffy *et al.* in which they performed a comprehensive screen of the Pathogen Box against multiple protozoan parasites including *T.b. brucei*.^17^ This current study and that of Duffy *et al.* used different criteria, from which to select compounds for dose-dependent analysis, therefore, in some instances an IC_50_ value may not have been determined for a compound from either study. However, a lack of an IC_50_ value does not necessarily mean that a compound was inactive, but rather that it was likely a moderate inhibitor. In addition, false positives and negatives are a burden of screening campaigns,^18^,^19^ therefore, for the purposes of robust analysis, we took this opportunity to compare our data with that obtained by Duffy *et al.* as well as that originally reported in the Pathogen Box. The majority of data we obtained in our study correlated well with that reported previously, particularly with respect to compounds from the kinetoplastid category of the Pathogen Box, which provided confidence in our method as well as for the previously reported data. However, inhibitory data for a small cohort of compounds from other categories of the pathogen differed to that reported by Duffy *et al.* Accordingly, we report our findings as an independent analysis of the Pathogen Box, which highlights potential new starting points for anti-trypanosomal drug discovery.

## Results and discussion

Prior to subjecting compounds to dose-dependent assessments, the Pathogen Box was screened for *T.b. brucei* inhibitory activity at a single concentration (10 μM). Compounds which inhibited cell viability to below 20% (*i.e.* reduced cell viability by 80% or more) at this concentration were put forward for dose dose-dependent assessment.

**Fig. 1:**
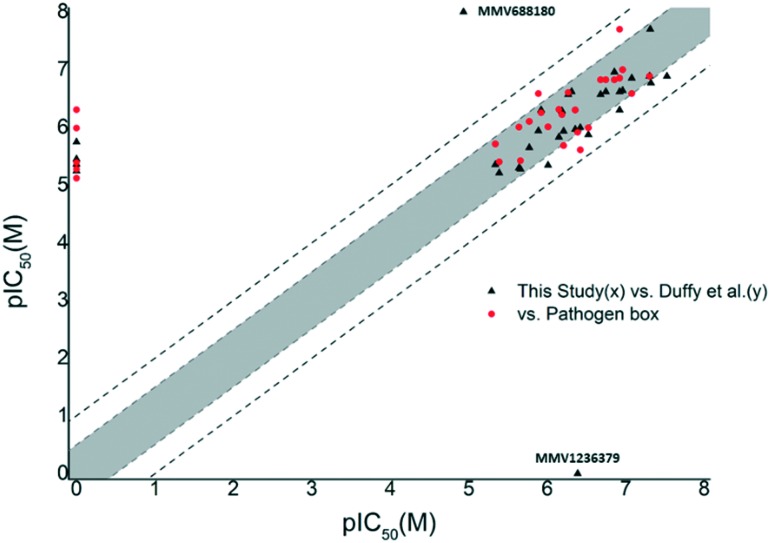
Plot of pIC_50_ (*T.b. brucei*) data obtained in this study (*x*) *vs.* the Pathogen Box or Duffy *et al.* (*y*) from compounds in the kinetoplastid category of the Pathogen Box. Compounds falling outside the grey shaded area differed in pIC_50_ by 0.5 log units or more. Compounds falling outside the dashed lines differed by 1 log unit or more. This figure indicated that our data was in good agreement with previous reports.


We opted for fairly stringent exclusion criteria, since the principal aim of this study was to identify or confirm compounds with activity in the sub-micromolar range. This data is supplied in the supplementary information. Of the 70 compounds categorised in the Pathogen Box as active against kinetoplastids, 34 were classified as inhibitors of *T.b. brucei*. Of these, 29 compounds (**1–22**, **25**, **28–31**, **33**, **34**) met our exclusion threshold and were subjected for IC_50_ evaluation (Table 1). In order to assess the robustness of our screen, we plotted pIC_50_ values (M) from the Pathogen Box *vs.* our experimentally obtained values (Fig. 1). Inhibitory data which was not provided as a precise value, but rather as an approximation below a threshold (*i.e.* <0.13 μM) was left out of our initial plot. Gratifyingly, this data showed that in all instances where IC_50_ data was obtained, the difference in pIC_50_ was lower than 1 (an order of magnitude). However, five compounds (**15**, **17**, **19**, **20**, **21**) differed by greater than 0.5 log units. Five compounds (**23**, **24**, **26**, **27**, **32**) appeared on the base on the *x*-axis, and represent compounds which did not sufficiently reduce cell viability in our single concentration screen, and were not subjected to IC_50_ analysis. However, of these compounds, only compound **24** (**MMV688776**) had activity in the sub-micromolar range reported in the Pathogen Box. The same plot was obtained by comparing the data of Duffy *et al.* and ours (Fig. 1). In this instance a change in pIC_50_ of greater than 0.5 log units was observed for six compounds (**1**, **4**, **7**, **15**, **21**, **29**) with only compound **1** (**MMV688180**) displaying a change in pIC_50_ greater than 1. However, this was a significant outlier. Compound **1** has previously been reported as a moderate inhibitor of *T. brucei* culture through the selective inhibition of *T. brucei N*-myristoyl transferase.^20^,^21^

**Table 1:** *In vitro* activity of the kinetoplastid category of the Pathogen Box against *T.b. brucei*

Cpd no.	ID	*T. brucei brucei* IC_50_ (μM)			HeLa IC_50_ (μM)
		Duffy *et al.*^*a*^	Pathogen Box^*b*^	This study	
**1**	MMV688180	0.01	<0.13	11	2.8
**2**	MMV688372	0.02	<0.13	0.049	0.8
**3**	MMV652003	0.11	0.15	0.14	>25
**4**	MMV688550	0.13	<0.13	0.030	>25
**5**	MMV688797	0.13	0.13	0.050	>25
**6**	MMV676604	0.14	0.26	0.084	1.2
**7**	MMV676602	0.17	<0.13	0.048	4.0
**8**	MMV688796	0.23	0.10	0.11	>25
**9**	MMV688371	0.24	<0.13	0.49	7.5
**10**	MMV688958	0.24	0.15	0.18	>25
**11**	MMV689028	0.24	0.14	0.12	>25
**12**	MMV675998	0.27	0.25	0.54	>25
**13**	MMV688795	0.27	0.15	0.21	>25
**14**	MMV688798	0.50	0.56	1.2	>25
**15**	MMV690027	0.50	0.02	0.12	>25
**16**	MMV688271	0.52	0.60	0.65	17
**17**	MMV202553	1.0	2.46	0.38	>25
**18**	MMV689029	1.1	0.50	0.44	>25
**19**	MMV688467	1.2	0.26	1.3	>25
**20**	MMV688793	1.2	2.07	0.62	>25
**21**	MMV676600	1.3	1.02	0.30	>25
**22**	MMV188296	1.5	0.49	0.71	>25
**23**	MMV687762	1.8	7.58	ND	ND
**24**	MMV688776	1.8	0.50	ND	ND
**25**	MMV690028	2.3	0.79	1.7	>25
**26**	MMV688514	3.5	4.06	ND	ND
**27**	MMV595321	4.3	5.30	ND	ND
**28**	MMV099637	4.4	1.94	4.6	>25
**29**	MMV688279	4.5	0.98	0.98	15
**30**	MMV687706	5.1	0.99	2.3	11
**31**	MMV688283	5.3	3.78	2.2	>25
**32**	MMV688179	5.7	1.03	ND	ND
**33**	MMV001561	6.2	3.97	4.1	10
**34**	MMV1236379	ND^*c*^	1.22	0.41	>25
	Pentamidine			0.0031	
	Emetine				0.4

^*a*^Data reported in ref. 17.

^*b*^Data available at https://www.pathogenbox.org/.

^*c*^ND = not determined.

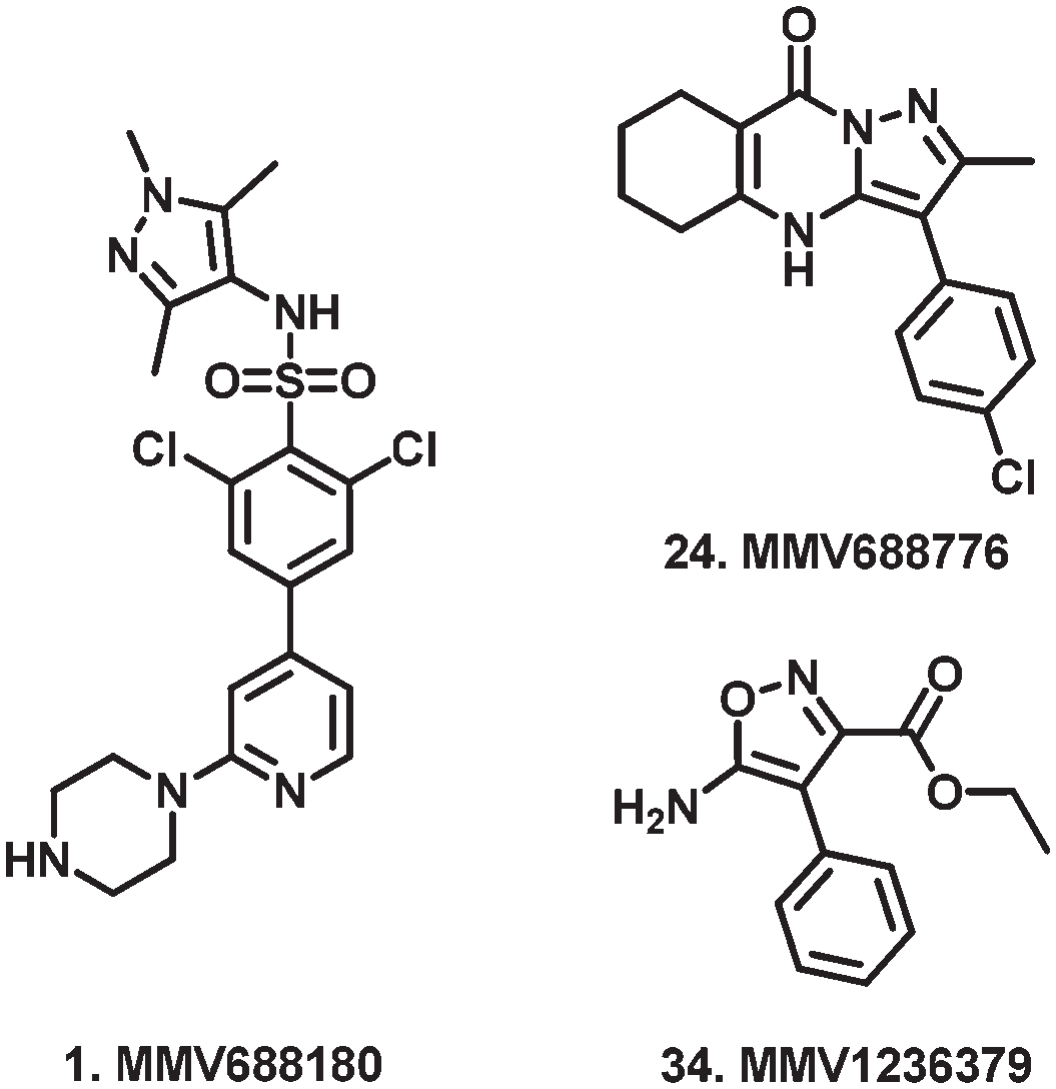
None of the other five compounds for which we obtained no IC_50_ values (**23**, **24**, **26**, **27**, **32**) inhibited *T.b. brucei* below 1 μM in Duffy *et al.*'s screen, and were not considered by us to have potent activity.

While Duffy *et al.* did not obtain an IC_50_ value for compound **34** (**MMV1236379**), our data suggested that **34** was a mid-nanomolar inhibitor of *T.b. brucei*, while the Pathogen Box placed it at the low micromolar range. While there were several instances, where compound potency differed by more than 0.5 log units, the comparative data obtained in this screen indicated that for the large majority of compounds, the pIC_50_ values we obtained fall within a 0.5–1 log range when compared to the Pathogen Box and Duffy *et al.* which we defined as an acceptable degree of similarity with previously reported data. With the exception of **1**, **24** and **34**, the majority of compounds whose data did not match to at least within an order of magnitude were compounds which were not subjected to dose-dependent analysis due to those compounds not conforming to our criteria in the single concentration screen (cell viability ≤20%). The data of Duffy *et al.* as well as the Pathogen Box, show that these were all low micromolar inhibitors, of *T.b. brucei* possibly accounting for their moderate performance in the single concentration screen, which indicated that overall, our screening method was robust. The correlation of our screening results and that of Duffy *et al.* highlights the complexities associated with the confirmation of biological activity and should also be seen in the context of differences in the detailed assay methodology employed. We used 96-well as opposed to 384-well plates, and compounds were incubated with parasites for 48 hours at 37 °C, while Duffy *et al.* added an additional 2 hours at 37 °C and 22 hours at room temperature.

From screening the remainder of the Pathogen Box listed for indications other than kinetoplastids, we identified 28 compounds (**35–62**), which satisfied our single concentration screening criteria, while several compounds (**63–69**) for which Duffy *et al.* obtained IC_50_ data, did not fit our criteria. (Table 2). The remainder of the Pathogen Box was inactive in both screens. Of compounds **63–69**, only the anti-mycobacterial **66** (**MMV687807**) whose MOA has been speculated to occur *via* disruption of mitochondrial proton gradient^22^ was reported by Duffy *et al.* as a sub-micromolar inhibitor of *T.b. brucei*. Similarly, we obtained IC_50_ data for 13 compounds (**38**, **39**, **43–47**, **49**, **53**, **55**, **59**, **60**, **62**), which were not reported by Duffy *et al.* likely due to their own cut off criteria of 5 μM in the primary screen. Of these compounds, **39** (**MMV676477**), **46** (**MMV687812**) and **53** (**MMV010576**) were identified as sub-micromolar inhibitors of *T.b. brucei*. The anti-mycobacterial compound **39** (ref. 23) has yet to be reported as an anti-trypanosomal. However, the closely related analogue TCMDC 142497 (**70**) was identified from a screen of 1.8 million compounds as a potent kinetoplastid inhibitor.^24^

**Table 2:** *In vitro* activity of the remaining Pathogen Box against *T.b. brucei*

Cpd no.	ID	*T. brucei brucei* IC_50_ (μM)			Selectivity index^*c*^
		Duffy *et al.*^*a*^	This study	HeLa IC_50_ (μM)	
**35**	MMV021013	3.51	0.83	>25	30
**36**	MMV024311	5.15	2.2	8.9	4.0
**37**	MMV153413	2.99	11	>25	2.2
**38**	MMV461553	ND^*b*^	11	>25	2.2
**39**	MMV676477	ND	0.59	2.6	4.4
**40**	MMV676411	3.22	0.76	8.4	11
**41**	MMV676512	2.87	1.1	5.1	4.6
**42**	MMV687273	2.28	2.3	15	6.5
**43**	MMV676409	ND	2.9	>25	8.6
**44**	MMV687703	ND	4.2	22	5.2
**45**	MMV687765	ND	5.1	>25	4.9
**46**	MMV687812	ND	0.87	4.8	5.5
**47**	MMV688844	ND	29	>25	0.8
**48**	MMV675968	2.07	1.2	>25	21
**49**	MMV687776	ND	1.2	10	8.3
**50**	MMV688417	6.10	4.3	>25	5.8
**51**	MMV024035	2.72	1.0	7.5	7.5
**52**	MMV006901	3.36	4.7	>25	5.3
**53**	MMV010576	ND	0.33	>25	76
**54**	MMV019189	4.68	1.0	6.6	6.6
**55**	MMV020391	ND	6.5	12	1.8
**56**	MMV022029	2.38	0.22	13	59
**57**	MMV023233	3.29	1.2	5.4	4.5
**58**	MMV028694	2.39	1.5	>25	17
**59**	MMV1030799	ND	7.1	>25	3.5
**60**	MMV002817	ND	2.0	>25	13
**61**	MMV688761	3.49	11	>25	2.3
**62**	MMV637229	ND	3.5	>25	7.1
**63**	MMV272144	4.12	ND	ND	
**64**	MMV495543	5.45	ND	ND	
**65**	MMV687248	1.05	ND	ND	
**66**	MMV687807	0.66	ND	ND	
**67**	MMV022478	1.45	ND	ND	
**68**	MMV026490	6.02	ND	ND	
**69**	MMV688768	1.50	ND	ND	
	Pentamidine		0.0031		
	Emetine			0.4	

^*a*^Data reported in ref. 17.

^*b*^ND = not determined.

^*c*^Selectivity index calculated as IC_50_(HeLa)/IC_50_(*T.bb*).


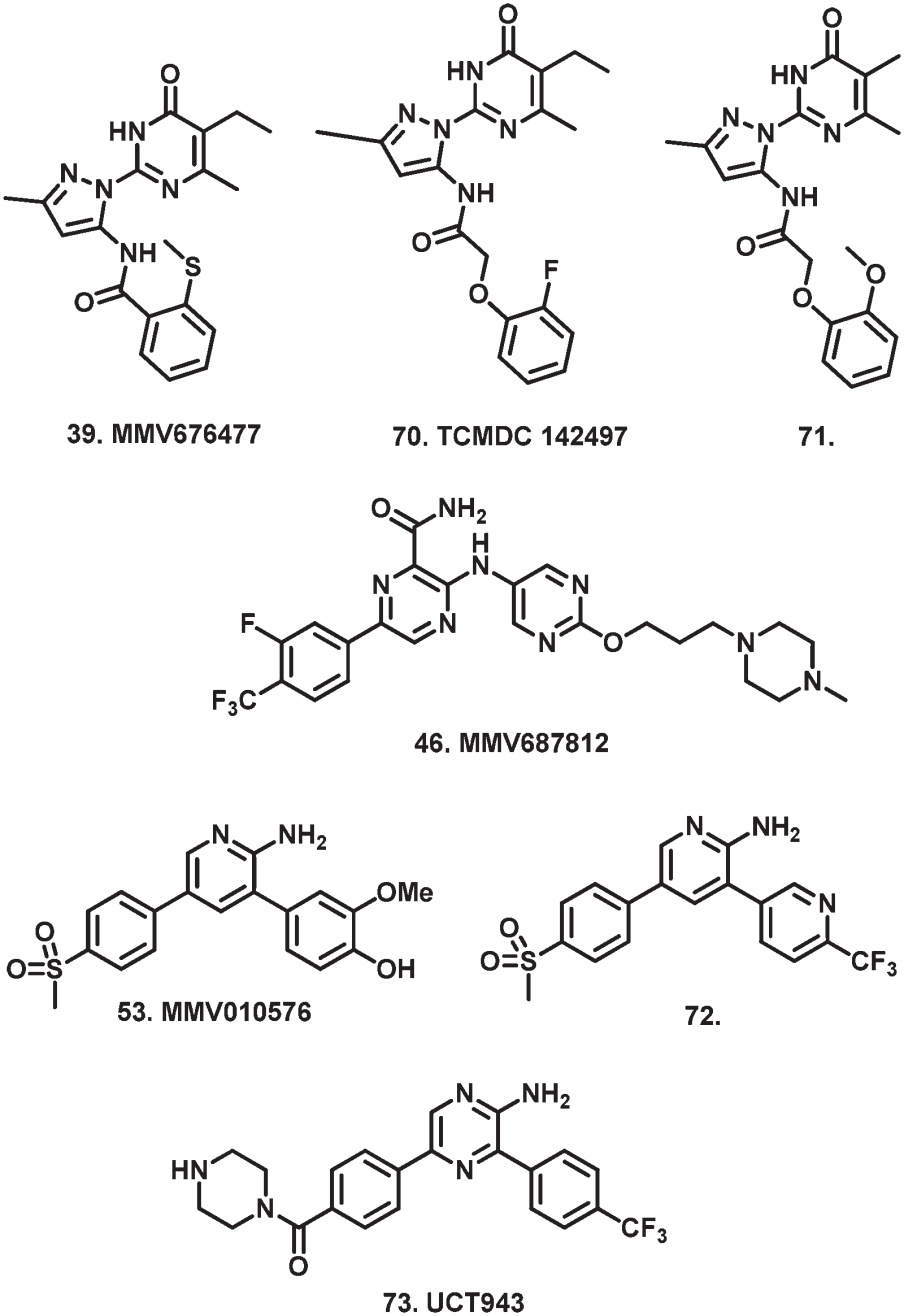
Another analogue (**71**) has been identified as an inhibitor of neuroblastoma, through the inhibition of the tyrosine kinase TrkB.^25^ Tyrosine kinases have been identified as potential targets for the inhibitions of *T. brucei*.^26^ Compound **46** was identified as a potent *in vitro* anti-mycobacterial which inhibits the ATPase domain of DNA gyrase B.^27^ Inhibition of DNA gyrase has been shown as an effective means of inhibiting kinetoplastid viability.^28^,^29^ The 3,5-diaryl-2-aminopyridine (**53**) has previously been identified as a potential inhibitor of *P. falciparum* transmission.^30^ Furthermore, it was found to be a potent and selective anti-plasmodial from a screen of the SoftFocus kinase library whose optimisation led to the identification of compound **72** which was curative in murine *P. berghei* following a single orally administered dose,^31^ and is currently undergoing clinical trials.^32^ Importantly, compound **72** was found to inhibit multiple malaria life stages, through its activity at *Plasmodium* phosphatidylinositol 4-kinase (PI4K), which is responsible for the regulation of intra- cellular signalling and trafficking through lipid phosphorylation.^33^,^34^ Trypanosomal PI4Ks have been shown to be essential for protein trafficking, Golgi structure, cytokinesis and normal cellular shape, and have been proposed as potential targets for trypanosomal drug discovery.^35^,^36^ Recently, further optimisation resulted in the identification of pre-clinical candidate **73** (UCT943), which is an improved multistage inhibitor of *P. falciparum*.^37^,^38^ Furthermore, Duffy *et al.* identified **53** as a moderate inhibitor of *Leishmaniasis donovani*.

**Fig. 2:**
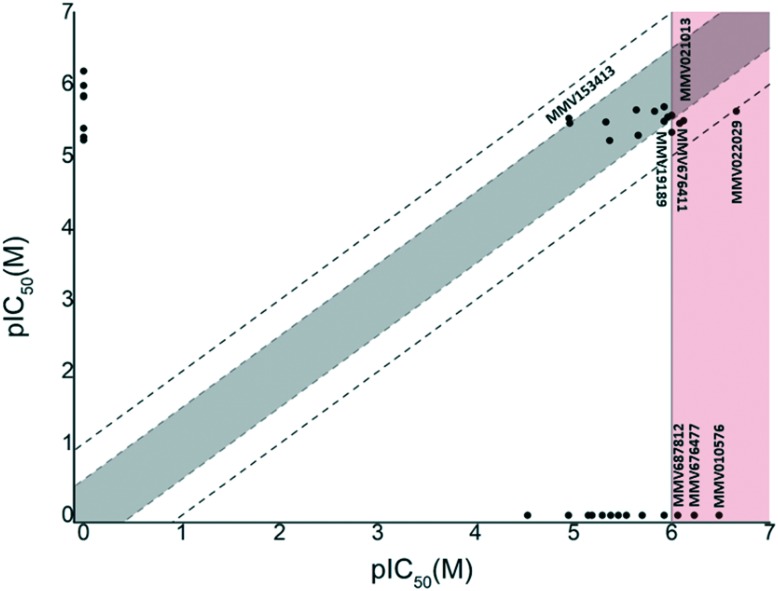
Plot of pIC_50_ (*T.b. brucei*) data obtained in this study (*x*) *vs.* Duffy *et al.* (*y*) from the reminder of the Pathogen Box. Compounds falling outside the grey shaded area differed in pIC_50_ by 0.5 log units or more. Compounds falling outside the dashed lines differed by 1 log unit or more. The pink shaded area highlights compounds which inhibited *T.b. brucei* in the sub-micromolar range in our screen. Compounds **35**, **39**, **40**, **46**, **53**, **54** and **56** were all found in this area and either differed 0.5 log units or more, or IC_50_ data was not determined for them by Duffy *et al.*


Comparison of remaining pIC_50_ values again showed that the majority of compounds did not differ by more than 0.5 log units from the values reported by Duffy *et al.* In our screen, the pIC_50_ of compound **37** (**MMV153413**) was found to be more than 0.5 log units lower than that reported by Duffy *et al.* (Fig. 2). However, our moderate inhibitory data for **37** matched that of its close analogue **38** (**MMV461553**), for which Duffy *et al.* did not obtain IC_50_ data. Both of these compounds specifically inhibit mycobacterial cell wall synthesis^39^ likely through the inhibition of mycolic acid biosynthesis.^40^ The remaining four compounds whose pIC_50_ values differed by more than 0.5 log units (**35**, **40**, **54**, **56**) were all found to have pIC_50_ values greater than six, corresponding to sub-micromolar inhibition. Compounds **35** (**MMV021013**)^23^ and **56** (**MMV022029**)^41^ had been identified as promising anti-mycobacterial and anti-plasmodial compounds respectively from large scale screening, without compelling evidence for a biological target.

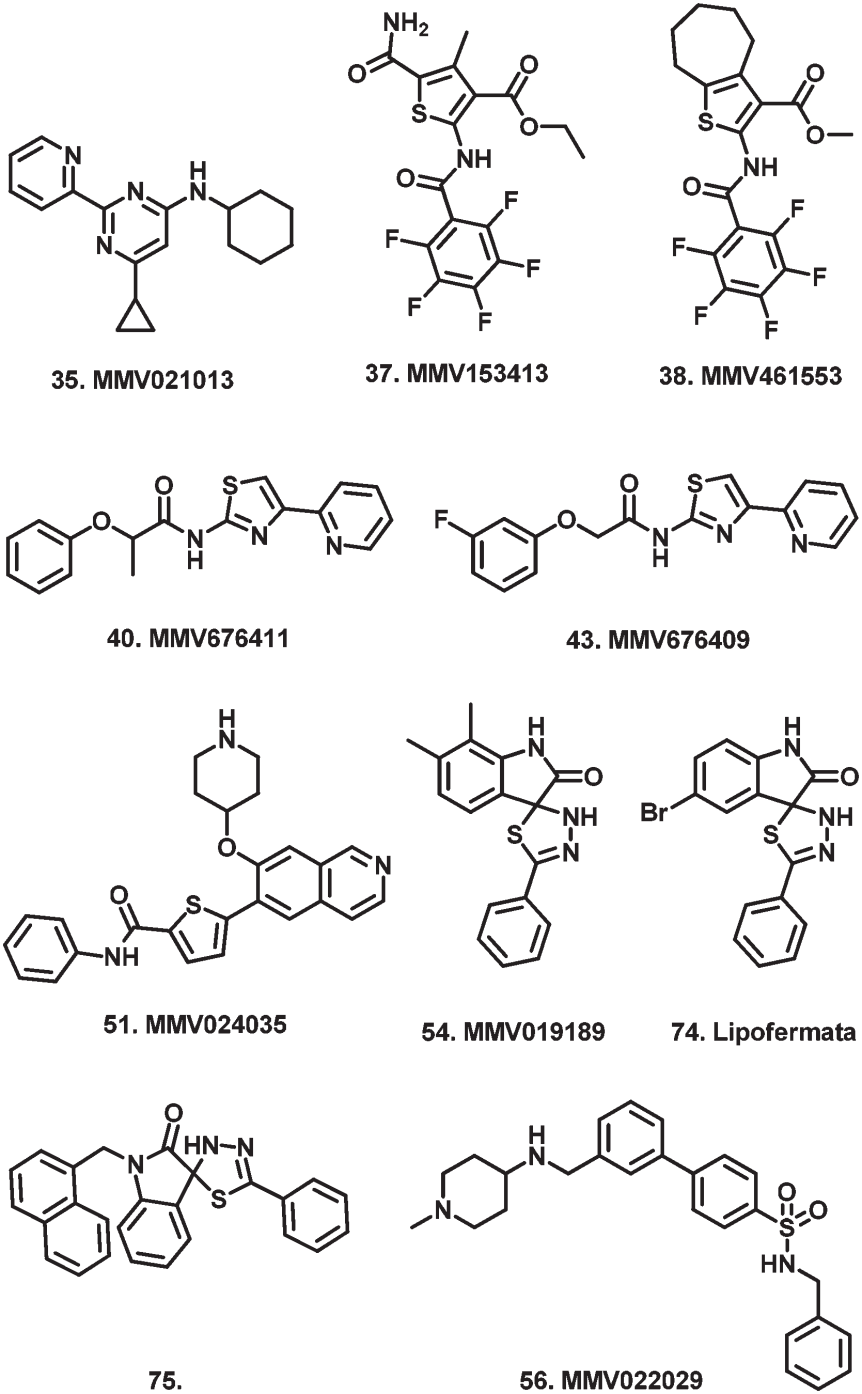
However, compound **40** (**MMV676411**) and its Pathogen Box analogue **43** (**MMV676409**) were identified as moderate inhibitors of *M. tuberculosis* CTP synthase, PyrG, which is responsible for the committed final step in pyrimidine biosynthesis.^42^ While trypanosomes possess similar machinery for the *de novo* synthesis of pyrimidines, they are also capable of pyrimidine salvaging, thereby limiting the use of this targeting strategy *in vivo*.^43^,^44^ However, this does not exclude the possibility of **40** inhibiting an alternative target.

Compound **54** (**MMV019189**) had also previously been reported as an anti-plasmodial agent.^41^ While to date there have been no reports of a potential mechanism of action for **54**, the structurally related compounds **74** (Lipofermata) and **75** have been identified as inhibitors of human fatty acid transport protein (FATP2)^45^ and metallo-β-lactamase.^46^ However, it is unclear whether analogous targets are present in *T. brucei*.

**Fig. 3:**
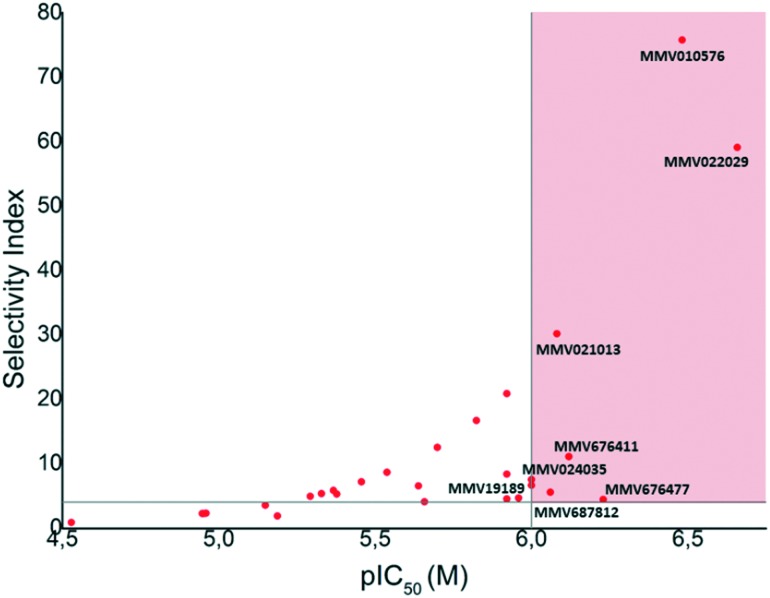
Plot of pIC_50_ (*T.b. brucei*) data obtained in this study (*x*) *vs.* selectivity index (*y*) from the reminder of the Pathogen Box. Compounds appearing in the pink shaded area combined sub-micromolar activity with acceptable selectivity over a HeLa cell line. Compounds **35**, **39**, **40**, **46**, **51**, **53**, **54**, and **56** were all deemed to be promising compounds for further study based on this assessment.

Having determined cytotoxicity against a HeLa cell line, we were able to derive the selectivity index (SI) for all active compounds (Table 2). A plot of pIC_50_*vs.* SI allowed us to rapidly identify which compounds combined good activity (pIC_50_ > 6) with acceptable selectivity (SI > 4) (Fig. 3). Compounds **35**, **39**, **40**, **46**, **53**, **54**, and **56** which have been discussed previously, all satisfied these criteria. Of these compounds, **39** and **46** are respectively reported to have CC_50_ values of 1.3 μM and 3.9 μM against HepG2 cells by the Pathogen Box, which agrees with our respective values of 2.6 μM and 4.8 μM against HeLa cells. While these compounds are very close to our selectivity index cut-off of 4, their encouraging activity may warrant further investigation and SAR analysis. None of the other compounds were reported as cytotoxic against HepG2 cells in the Pathogen Box dataset. Furthermore, antimalarial compound **51**^41^ (**MMV024035**) was identified as a candidate that selectively inhibited *T.b. brucei*, albeit moderately. This compound also showed moderate inhibition in the screen of Duffy *et al.*

## Conclusions

In conclusion, this study sought to identify new starting points for anti-trypanosomal drug discovery, through the repurposing of the Pathogen Box. Our results have identified eight compounds, which may hold promise as starting points for in-depth SAR studies. Compounds **35**, **53** and **56** in particular combined activity in the nanomolar range coupled to good selectively, while compounds **39**, **46** and **53** again hold potential for target based programs due to their reported activity against targets for which analogous biological processes are present in *T. brucei*.

## Experimental procedures

### *Trypanosoma brucei* assay

*T.b. brucei* (strain 427) bloodstream form parasites were cultured at 37 °C in a 5% CO_2_ incubator in IMDM medium supplemented with 25 mM HEPES, 10% fetal bovine serum, 1 mM hypoxanthine, antibiotics (penicillin/streptomycin) and HMI-9 supplement.^47^ For screening purposes, parasites were distributed with\ test compounds in 96-well plates at a final concentration of 10 μM compound and 2.4 × 10^4^ parasites per well in a total volume of 200 μl per well. After a 24 hour incubation at 37 °C in a 5% CO_2_ incubator, 20 μl resazurin reagent (0.5 mM resazurin in phosphate-buffered saline) was added to each well and incubation continued for a further 24 hours. Resazurin conversion to resorufin was determined by measuring fluorescence (Exc_560_/Em_590_) in a plate reader. Fluorescence values obtained in drug-treated wells were converted to % parasite viability relative to readings obtained in control wells (non-treated parasites). Dose–response assays were performed by incubating parasites with 3-fold serial dilutions of test compounds, plotting % parasite viability *vs.* log[compound] and determining IC_50_ concentrations by non-linear regression analysis using GraphPad Prism. All assays were performed in technical triplicate.

### HeLa cell assay

HeLa cells were cultured in DMEM medium supplemented with 10% fetal bovine serum and antibiotics (penicillin/streptomycin/amphotericin B) at 37 °C in a 5% CO_2_ incubator. On the day prior to compound addition, cells were plated in 96-well plates at 2 × 10^4^ cells per well. Compounds were added to the cells in 3-fold serial dilutions and incubation at 37 °C in a 5% CO_2_ incubator continued for 48 hours. Twenty μl resazurin reagent (0.5 mM resazurin in phosphate-buffered saline) was added to each well and, after a 2-hour incubation, fluorescence (Exc_560_/Em_590_) was measured in a plate reader. Percentage cell viability in drug-treated wells was calculated from the fluorescent readings obtained relative to those in wells containing control, untreated cells. Plots of % cell viability *vs.* log[compound] were used to determine IC_50_ values by non-linear regression analysis using GraphPad Prism.

## Conflicts of interest

There are no conflicts to declare.

## Supplementary Material

Supplementary information
